# A Structural Switch between Agonist and Antagonist Bound Conformations for a Ligand-Optimized Model of the Human Aryl Hydrocarbon Receptor Ligand Binding Domain

**DOI:** 10.3390/biology3040645

**Published:** 2014-10-17

**Authors:** Arden Perkins, Jessica L. Phillips, Nancy I. Kerkvliet, Robert L. Tanguay, Gary H. Perdew, Siva K. Kolluri, William H. Bisson

**Affiliations:** 1 Department of Biochemistry and Biophysics, Oregon State University, Corvallis, OR 97331, USA; E-Mail: perkina2@eou.edu; 2 Cancer Research Laboratory, Corvallis, OR 97331, USA; E-Mail: philljes@onid.orst.edu; 3 Department of Environmental and Molecular Toxicology, Environmental Health Sciences Center, Oregon State University, Corvallis, OR 97331, USA; E-Mails: nancy.kerkvliet@oregonstate.edu (N.I.K.); robert.tanguay@oregonstate.edu (R.L.T.); 4 Center for Molecular Toxicology and Carcinogenesis, Department of Veterinary and Biomedical Sciences, The Pennsylvania State University, University Park, PA 16802, USA; E-Mail: ghp2@psu.edu

**Keywords:** aryl hydrocarbon receptor, ligand binding domain, agonist, antagonist, ligand-guided optimization, virtual ligand screening, molecular dynamics, HSP90

## Abstract

The aryl hydrocarbon receptor (AHR) is a ligand-activated transcription factor that regulates the expression of a diverse group of genes. Exogenous AHR ligands include the environmental contaminant 2,3,7,8-tetrachlorodibenzo-p-dioxin (TCDD), which is a potent agonist, and the synthetic AHR antagonist *N*-2-(1H-indol-3yl)ethyl)-9-isopropyl-2-(5-methylpyridin-3-yl)-9H-purin-6-amine (GNF351). As no experimentally determined structure of the ligand binding domain exists, homology models have been utilized for virtual ligand screening (VLS) to search for novel ligands. Here, we have developed an “agonist-optimized” homology model of the human AHR ligand binding domain, and this model aided in the discovery of two human AHR agonists by VLS. In addition, we performed molecular dynamics simulations of an agonist TCDD-bound and antagonist GNF351-bound version of this model in order to gain insights into the mechanics of the AHR ligand-binding pocket. These simulations identified residues 307–329 as a flexible segment of the AHR ligand pocket that adopts discrete conformations upon agonist or antagonist binding. This flexible segment of the AHR may act as a structural switch that determines the agonist or antagonist activity of a given AHR ligand.

## 1. Introduction

**Figure 1:**
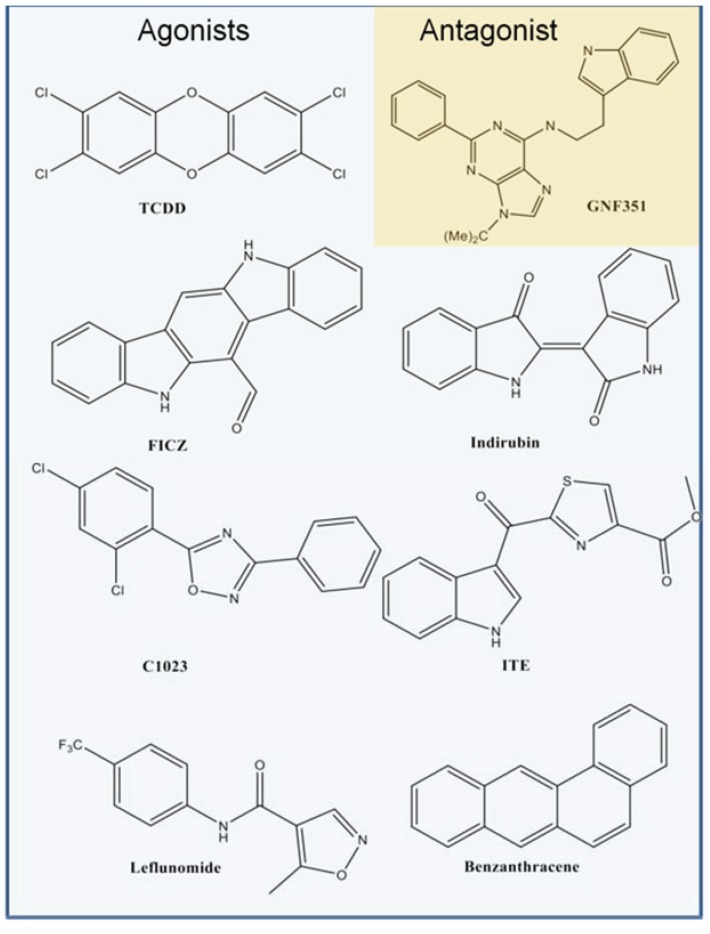
Chemical structures of aryl hydrocarbon receptor (AHR) ligands. The chemical structures of selected agonists from the benchmarking database are shown in blue, and the structure of antagonist GNF351 is shown in orange.

The aryl hydrocarbon receptor (AHR) is a ligand-activated transcription factor that regulates the expression of a diverse group of genes, including members of the drug-metabolizing P_1_-450 family, aldehyde dehydrogenase 3, and prostaglandin endoperoxide H synthase-2, and is involved in cross-talk with the inflammatory signaling and estrogen receptor pathways [1,2,3]. Latent AHR is cytosolic and forms a complex with heat shock protein 90 (HSP90) [1], X-associated protein, and p23 [4]. Upon binding of an agonist to AHR, the complex translocates to the nucleus and AHR dissociates from the chaperone proteins and heterodimerizes with the AHR nuclear translocator (ARNT). The AHR-ARNT complex subsequently binds to its DNA recognition site and initiates transcription of target genes [1]. Investigations into the AHR ligand-binding pocket have in part been motivated by the goal of identifying molecules that bind AHR and elicit distinct biological functions. Molecules with a diverse array of chemical scaffolds have been found to modulate AHR activity [5,6,7]. For example, the environmental contaminant 2,3,7,8-tetrachlorodibenzo-p-dioxin (TCDD, Figure 1) is one of the strongest agonists of the AHR [1,8], and high throughput screening (HTS) methods recently drove the characterization of the FDA approved drugs leflunomide and raloxifene as new human AHR agonists [9,10]. Alternatively, antagonists such as *N*-2-(1H-indol-3yl)ethyl)-9-isopropyl-2-(5-methylpyridin-3-yl)-9H-purin-6-amine (GNF351, Figure 1) have been characterized that block agonist-induced AHR transcriptional activity [7]. GNF351 was originally discovered through HTS techniques [5], and recent studies have revealed its anti-inflammatory effects [11,12]. Thus, the identification of AHR ligands helps to expand our understanding of the function of this transcription factor and aids in AHR-based therapeutic design.

The AHR consists of (in order from *N*-terminus to *C*-terminus) a basic helix-loop-helix domain, two Per/ARNT/Sim (PAS, defined as A and B) domains, and a transactivation domain [1]. Especially of interest is the PAS-B domain (residues ca. 278–390), as it functions as the ligand-binding domain and has been shown in the murine system to constitute a major interface with HSP90 [1]. Insights into the molecular structure of the ligand-binding domain can potentially elucidate AHR function and aid in the prediction of new ligands that modulate the AHR. However, no experimentally determined structure of the AHR ligand-binding domain structure is available. Therefore, structural studies have been primarily driven by homology modeling [6,13,14]. These models are based on the PAS-B domain of hypoxia-inducible factor 2α (HIF-2α), which exhibits the closest sequence identity (ca. 30%) to the AHR PAS-B domain, and its structure has been determined by nuclear magnetic resonance (NMR) [15] and X-ray crystallography [16]. Homology models of the AHR PAS-B domain, coupled with mutagenesis studies, have been useful to address species selective differences in TCDD binding [6,17] and to identify potential binding patterns of agonists and antagonists of the AHR [6,13,18,19]. Homology models have also assisted in the identification of novel ligands through Virtual Ligand Screening (VLS), by docking and ranking thousands of candidate compounds for HTS testing [6].

Here, we present a new strategy for generating AHR homology models with an enhanced ability for identifying agonists. We docked the strong agonist TCDD into our *apo* human AHR PAS-B binding pocket [6] and optimized side chain orientations to accommodate the ligand to create an “agonist-optimized” model. We hypothesized that this method would generate a binding pocket more representative of the agonist-bound state and therefore improve agonist identification during molecular docking. When applied to a large-scale VLS campaign, this agonist-optimized model aided in the identification of novel agonists that were confirmed in cell-based assays. In addition, we have conducted molecular dynamics simulations of the TCDD-bound and GNF351-bound AHR PAS-B conformations, and we observed distinct structural changes induced by agonist and antagonist interactions that may play a role in switching between agonist and antagonist activity.

## 2. Experimental Section

### 2.1. Homology Modeling

HIF-2α has the highest sequence similarity to the human AHR PAS-B (ca. 30%) for which an experimental structure has been determined and has been used as a template structure for AHR homology model generation in several previous studies [6,13,14,17,19]. We considered two structures of HIF-2α PAS-B which might be used as templates for a human AHR homology model: an apo NMR structure (1p97, [15]) and a 1.5 Å holo crystal structure with N-(furan-2-ylmethyl)-2-nitro-4-(trifluoromethyl)aniline bound in the pocket (PDB code 3h82, [20]). Our previous apo model of human AHR PAS-B was based on the NMR ensemble [15], but interspecies models of the AHR PAS-B using ligand-bound crystal structures of HIF-2α as templates were also published [13,14]. Since both structures could potentially produce useful homology models, we generated one ligand-optimized model based on the apo NMR ensemble and a second based on the holo crystal structure using the program Molsoft ICM [21].

Briefly, the sequences of the template structures were aligned with human AHR PAS-B as described previously [6], and preliminary models were generated with the structure of the conserved regions retained from the template. Using rigid docking with ICM (with the protein atoms fixed in position but the ligand allowed to be flexible) the strongest known agonist TCDD was docked into the NMR- and crystal-based models. The docking was conducted as previously reported [19]. In the docking method five types of interaction potentials represent the receptor pocket: (i) van der Waals potential for a hydrogen atom probe; (ii) van der Waals potential for a heavy-atom probe (generic carbon of 1.7 Å radius); (iii) optimized electrostatic terms; (iv) hydrophobic terms; and (v) loan-pair-based potential, which reflects directional preferences in hydrogen bonding [22]. For the ligand-optimization step of the NMR-based model, side chains within 4 Å of the TCDD docked pose were subjected to a fast 1×10^5^ step ICM Monte Carlo simulation and the complex calculated to be the most energetically favorable was used for VLS [23]. Throughout the process, geometrical quality of the models were monitored using the tool Protein Health implemented in ICM to calculate the relative energy strain for each residue [24]. When we superimposed the final NMR-based (ligand-optimized) and crystal-based homology (not ligand-optimized) models we noted that the binding pocket of the latter possessed a slightly different shape and was larger in volume (383 Å^3^ for the crystal-based *versus* 314 Å^3^ for the NMR-based). However, both pockets are near the ca. 400 Å^3^ range observed for the three previously reported *holo* crystal structures of HIF-2α PAS-B [13,16]. This therefore provided a preliminary indication that ligand-optimization could expand the binding pocket to a volume similar to that seen in experimental structures of *holo* PAS-B domains.

### 2.2. Virtual Ligand Screening

Receiving Operating Characteristics (ROC) curves and early enrichment measure are useful methods to validate models by testing a model’s ability to discriminate true binders from decoy compounds through VLS [22]. In our case, we could not build reliable ROC curves due to the limited number of known diverse AHR ligands. In order to assess the quality of our ligand-optimized models for the purpose of VLS, we performed three rounds of rigid docking against: (a) a decoy database of 5000 randomly chosen compounds from the commercially-available Chembridge Diversity Library in the 378–397 Da range; and (b) a benchmark database of forty-two molecules including sixteen known agonists of the human AHR [1,6,18,24,25,26,27] and twenty-six known human AHR “inactive” compounds, which we define here as compounds which show no agonist activity [6,28]. The results of the decoy database docking were used to calculate the threshold score for the top ranking 50 compounds (1%) of −5.314 (NMR-based model) and −30.68 (crystal-based model). Usually, lower scores predict better binding. The hit rate, calculated as the percentage of the known AHR agonists which docked with a score ranking above the threshold value, was 88% (14/16) for the NMR-based model and only 6.2% (1/16) for the crystal-based. We noticed that one critical difference in the crystal-based model that may have contributed to its lower hit rate was that the pocket conformation was such that a hydrogen bonding interaction between the known agonists and the side chain of Ser365 was never detected. Due to the poor ability of the crystal-based homology model to distinguish known agonists from random compounds we chose to proceed only with the NMR-based model for our studies. For clarity, all further discussion will refer explicitly to the NMR-derived agonist-optimized homology model of AHR PAS-B.

The agonist-optimized benchmarking results placed TCDD with the top score (−24, Table 1). To utilize the agonist-optimized model for the purpose of identifying novel agonists, we next performed VLS with the Chembridge Diversity Library (ca. 50,000 compounds), using the same docking parameters as in the benchmarking calculation. We selected the top 500 compounds (1%) ranked by score and re-screened this subset database with three independent runs. The visual analysis of the three runs started from the top score compound down to −23, considering as criteria for ranking and selection both docking score and key TCDD-like manner interactions like hydrogen bonds to either His291 and/or Ser365. These residues are predicted to constitute part of the binding pocket, and have been shown to be critical for agonist binding (see below). Based on these criteria, sixteen compounds were chosen for further testing (Table 2).

**Table 1:** Docking results from the Virtual Ligand Screening (VLS) of the benchmark database into the agonist-optimized model ^a,b^.

Compound [Ref]	Score	Compound [Ref]	Score
**TCDD [8]**	**−24.00**	**Indole sulphate [26]**	**−11.33**
ST057251 [6]	−21.35	ST056006 [6]	−11.18
**C1023 [27]**	**−21.30**	ST056008 [6]	−11.07
**ST066905 [6]**	**−20.70**	ST056001 [6]	−10.62
**Leflunamide [9]**	**−20.15**	**ST056012 [6]**	**−10.17**
**ST069348 [6]**	**−18.33**	**ST057541 [6]**	**−9.73**
**ST055658 [6]**	**−16.62**	**FICZ [6]**	**−9.07**
**Benza(a)anthracene [1]**	**−16.60**	**ST023293 [6]**	**−9.00**
**ITE [6]**	**−15.57**	ST057152 [6]	−7.86
**Indirubin [25]**	**−11.35**	ST057260 [6]	−7.14
		ST056284 [6]	−6.18

^a^ Shown in bold are the known AHR activators with relative reference in the literature; only molecules scoring above the threshold value are listed. ^b^ “ST” indicates compounds commercially available at TimTec (www.timtec.net/).

**Table 2:** VLS docking score results of compounds selected for cell-based screening.

Compound ID ^a^		Score		Compound ID	Score
**D1**	64002551	−24.67	**D9**	83726764	−24.55
**D2**	71516112	−23.62	**D10**	5687214	−22.29
**D3**	54274015	−23.74	**D11**	99650393	−28.21
**D4**	86843844	−23.60	**D12**	20614137	−26.02
**D5**	64928850	−23.83	**D13**	5484675	−23.08
**D6**	17712589	−23.86	**D14**	22292121	−25.29
**D7**	34150640	−23.09	**D15**	68493444	−25.39
**D8**	65277029	−24.41	**D16**	93043217	−24.90

^a^ Refers to the compound name commercially available at ChemBridge (www.chembridge.com).

### 2.3. Cell-Based Screening

The sixteen compounds identified by VLS were purchased from Chembridge (San Diego, CA, USA), dissolved in dimethyl sulfoxide (DMSO), and tested in triplicate at a final concentration of 10 µM. The structures and ≥99% purity of the tested compounds were confirmed by Chembridge using liquid chromatography/mass spectrometry (LC/MS). All cells were cultured in Dulbecco’s Modified Eagle Medium (DMEM) with L-glucose, L-glutamine, and sodium pyruvate (Mediatech Inc., Manassas, VA, USA) supplemented with 10% fetal bovine serum (FBS) (Tissue Culture Biologicals, Long Beach, CA, USA), 100 IU/mL penicillin, and 100 µg/mL streptomycin (Mediatech Inc., Manassas, VA, USA) in a humidified 5% CO_2_ atmosphere. Cells were typically passaged every two days at a dilution of 1:4. For transient transfections in human HepG2 cells, the xenobiotic response element (XRE)-mouse mammary tumour virus (MMTV)-Luc expression vector was used. XRE-MMTV-Luc (hereafter referred to as XRE-Luc) contains a synthetic XRE oligonucleotide upstream of the MMTV viral promoter. The β-galactosidase expression vector, which expresses the β-galactosidase gene under control of a minimal cytomegalovirus (CMV) promoter (pCH 110; Pharmacia), was used for normalization of luciferase activity. PCDNA3.0 (Invitrogen, Grand Island, NY, USA) was used as carrier DNA for transfection normalization purposes. HepG2 cells were plated at a density of 0.75 × 10^5^ cells/well in 24 well plates and grown overnight. The following day the cells were transfected with 600 ng of the XRE-Luc expression vector, 100 ng β-galactosidase expression vector, and 300 ng PCDNA3.0 as carrier DNA using Lipofectamine 2000 (Invitrogen, Grand Island, NY, USA) according to the manufacturer's recommended protocol. Co-transfection with the β-galactosidase expression vector was used for normalization purposes. Approximately 24 h after transfection, the media was removed and the cells were treated with DMSO, 1 nM TCDD, or 10 µM of compounds for an additional 24 h; the total concentration of DMSO in experiments did not exceed 0.1% v/v. After incubation with the compounds, treatment media was removed and cells were lysed using 1 × Lysis Buffer (Promega) to a volume of 150 µL. 100 µL of lysate was assayed well-by-well for luciferase activity by injection of luciferase assay substrate (Promega) with a 2 s mixing time and 15 s integration period on a Tropix TR717 microplate luminometer. Twenty-five microliters of lysate were used for human β-galactosidase assays for transfection normalization. Beta-galactosidase activity was determined by incubating the cell lysates for approximately 20 min with 100 µL β-galactosidase reaction buffer per well (100 mM sodium phosphate buffer pH 7.3, 1.25 mM MgCl_2_, 62.5 mM 2-mercaptoethanol, and 1.1 mg/mL *O*-nitrophenyl-beta-d-galactopyranoside) at 37 °C, and measuring the absorbance at 405 nm in a spectromax 96-well plate reader (SpectraMax). To normalize the data in transient transfection experiments, raw luciferase values were divided by their respective β-galactosidase activity.

For analysis of AHR target gene induction in a dose-dependent manner, the human HepG2 hepatoma cell line was used. RNA was extracted from cells treated for 12 h with the compounds at 10 μM, 5 µM, and 1 µM or a vehicle dose of DMSO using the E.Z.N.A Total RNA Kit I (Omega Bio-Tek, Norcross, GA USA), and cDNA was synthesized using the SuperScript III First-Strand Synthesis System for reverse transcription-polymerase chain reaction (RT-PCR) (Invitrogen). RT-PCR was performed using ABI 7500 Fast instrument (ABI) and RT2Real-Time SYBR Green PCR Master Mix (SA Biosciences, Valencia, CA, USA). For analysis of AHR translocation to the nucleus upon activation by a ligand, cells were plated in a 96-well plate (µCLEAR, Greiner Bio One, Monroe, NC, USA) pre-coated with poly-d-lysine, cultured overnight, and then treated for 2 h with 10 µM of respective compounds.

Immunocytochemistry was performed using AHR antibody at a dilution of 1:1000 (Enzo) coupled with a Cy3 secondary antibody and ProLong Gold DAPI nuclear stain (Molecular Probes, Eugene, OR, USA). Images were collected by fluorescent microscopy via ImageXpressMICRO wide-field high content imaging system and were processed with MetaXpress version 3.1 software (Molecular Devices). Compounds **D12** and **D16** were able to strongly activate the endogenous target gene of the AHR *CYP1A1* and also promoted nuclear translocation of endogenous AHR. **D12** ([2-fluoro-5-(5-methyl-1,3-benzoxazol-2-yl) phenyl]methanol), which in VLS docked with a score of −26.02 (Table 2), was identified as the strongest AHR agonist in terms of both *CYP1A1* activation and AHR nuclear translocation.

### 2.4. Molecular Dynamics

Three versions of the agonist-optimized homology model were used as the starting coordinates for molecular dynamics simulations: (1) *apo* (the agonist-optimized homology model absent of TCDD); (2) agonist-bound (the agonist-optimized homology model with TCDD in the binding pocket); and (3) antagonist-bound (GNF351 docked into the agonist-optimized model). The prep files of the docked complexes were generated using the program ANTECHAMBER [29]. The models were solvated in a box of water molecules (TIP3P model) and Cl^−^ counter ions were added to the solvent to maintain neutrality of the system using LEAP [30] with periodic boundary conditions applied. The total number of atoms contained in the *apo*, agonist, and antagonist models were 26777, 28176, and 26833, respectively. All simulations were conducted with AMBER12, applying the parm99SB AMBER force field [31].

The models were first energy-minimized by 1000 cycles of steepest descent followed by the conjugate gradient method until the rmsd of the Cartesian elements of the gradient reached a value smaller than 0.15 Å. This was followed by three molecular dynamics steps, all utilizing a 0.002 ps time step, with initial velocities at the beginning of each simulation assigned from the Maxwellian distribution at the desired temperature, and cutoff distance for non-bonded interactions set to 9 Å. The first step (conducted at 0° K) was a short 15 ps equilibration of water molecules and ions around the fixed protein, with the trajectories of the water and ions allowed to evolve over time according to Newtonian laws. Second, (conducted at 150° K) a 15 ps equilibration at constant volume was performed on the entire system to adjust the density to 1 g/cm^3^. In the final stage (fixed at 300° K according to Berendsen’s coupling algorithms [30]) a 50 ns molecular dynamics simulation was performed at constant pressure of 1 atm without any constraints for conformational stability. After 50 ns, we judged the three simulations to have reached equilibration when we observed a plateau of the all-protein atom rmsd for ca. 5 ns and if no major shift in the conformation of protein or ligand had occurred for ca. 10 ns. Consequently, the *apo* was simulated for an additional 10 ns (totaling 60 ns) in order for that system to reach equilibration. Though the antagonist-bound simulation exhibited a slight decrease in all-atom rmsd near the end of the 50 ns simulation, comparing the model at 40 ns and 50 ns showed that the protein and ligand conformations were essentially unchanged, and therefore we judged the system to be equilibrated.

## 3. Results

### 3.1. Agonist-Optimization of the Human AHR PAS-B Model: Comparison between Models, Docking Results, and Experimental Validation of VLS Hits

**Figure 2:**
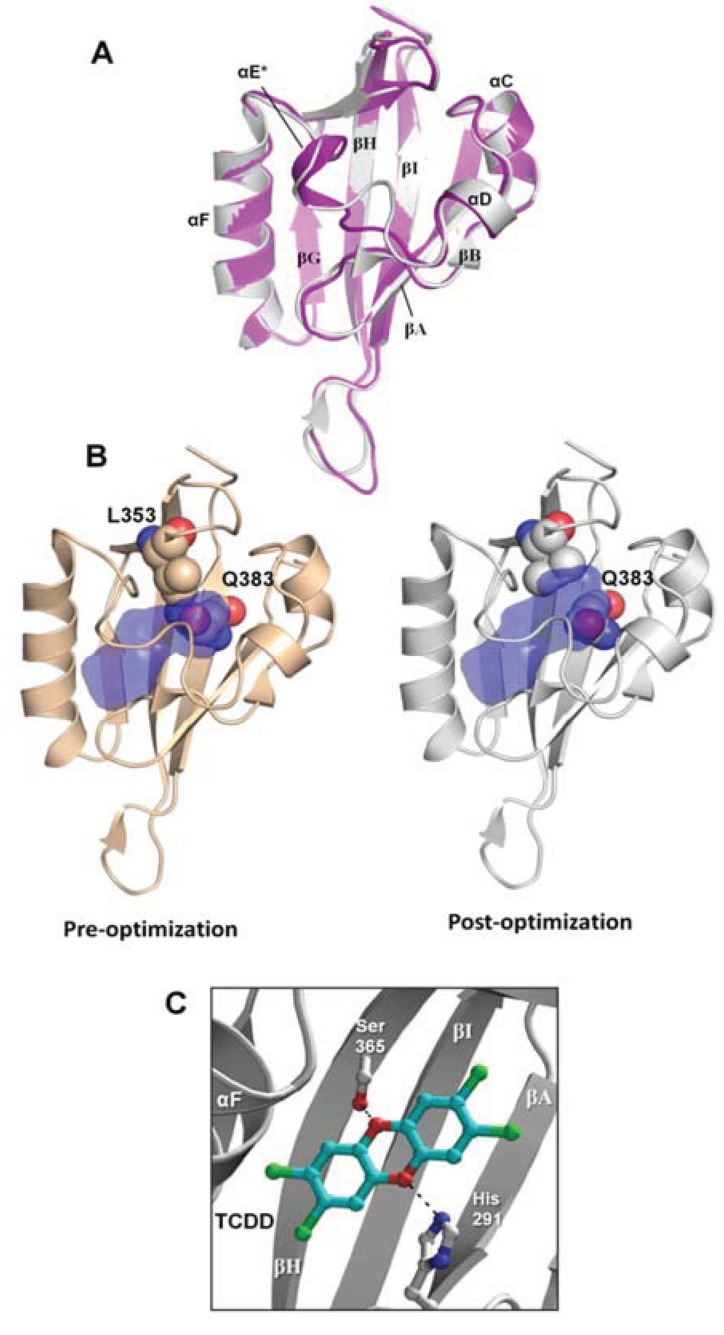
Agonist-optimized model of human AHR PAS-B. (**A**) Comparison of the agonist-optimized model (grey) with its template structure hypoxia inducible factor 2α (HIF-2α) Per/ARNT/Sim (PAS)-B (purple, PDB code 1p97). Standard PAS-B secondary structures are labeled. The helix αE is distinguished with an (*) because it adopts nearly helical geometry in the agonist-optimized model. The highest energy strain in the agonist-optimized model occurs at residues Phe324 (αE), Leu370 (βH), and Ile380 (βI). (**B**) The AHR PAS-B homology models pre-optimization and post-optimization are shown. The binding pocket is displayed in blue, with the pocket calculated as the solvent-accessible surface area using a spherical probe with a radius of 1.4 Å. Residues Leu353 and Gln383 are displayed as spheres and colored with oxygens red, nitrogens blue, and carbons brown or grey. (**C**) The docking orientation of 2,3,7,8-tetrachlorodibenzo-p-dioxin (TCDD) in the agonist-optimized model is shown, which forms hydrogen bonds to Ser365 and His291.

With the goal of identifying novel agonists of the human AHR, we sought to create a homology model of the ligand-binding domain that could complement HTS methods by efficiently and accurately screening large and chemically diverse libraries. As described in the Experimental section, we generated a homology model for the human AHR PAS-B domain based on an experimentally-determined structure of HIF-2α [15], docked the strong agonist TCDD into the binding pocket, and subsequently optimized the protein side chains near the ligand to search for an energetically-favorable complex. The resulting model, which we will refer to as the “agonist-optimized” model throughout the text, deviates from the template HIF-2α structure by 0.53 Å root-mean-square-deviation (rmsd) over 109 Cα atoms and the main differences in chain path occur at αE and the βH-βI loop (Figure 2a). As in our previous homology model of the *apo* AHR PAS-B [6], our agonist-optimized model contains the standard secondary structural elements of PAS-B domains: four α-helices and five β-strands denoted (in order from *N*-terminus to *C*-terminus) βA, βB, αC, αD, αE, αF, βG, βH, and βI (Figure 2a) [6]. The agonist-optimized homology model revealed an increase of 105 Å^3^ in the binding pocket volume compared to the *apo* model (Figure 2b) [6]. This difference is principally due to the movement of the Gln383 and Leu353 side chains, which in the agonist-optimized model expanded the cavity toward αC and αD (Figure 2b). Importantly, this made the pocket more accessible for the docking of larger ligands during VLS, and with this agonist-optimized model we were able to obtain docking scores [22] for a benchmarking of known AHR agonists (Table 1).

**Figure 3:**
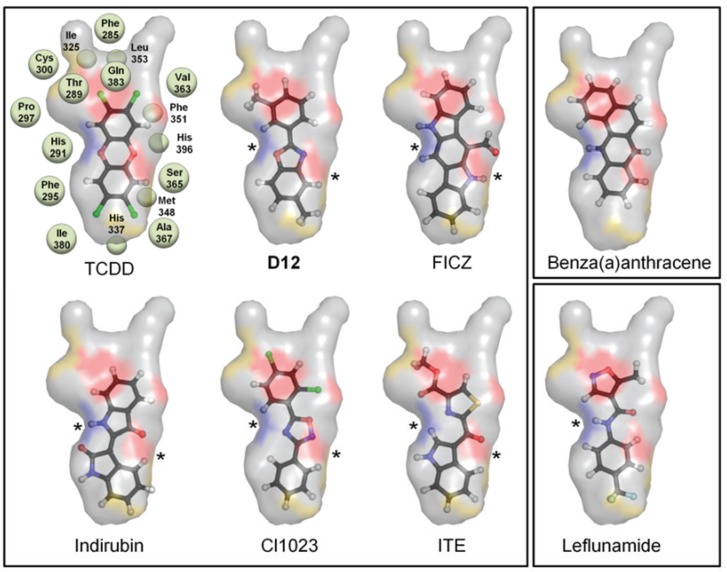
Binding pattern of AHR agonists. The top-scoring poses of selected AHR agonists docked into the agonist-optimized model are shown, with the pocket surface area contributed by the protein shown as transparent surface and calculated as in Figure 2. Atoms are colored by type (hydrogen-white, carbon-grey, oxygen-red, nitrogen-blue, sulfur-yellow, halogen-green). The residues constituting the pocket are indicated as grey spheres for TCDD. Some compounds shown docked in a TCDD-like manner, with the ability to form hydrogen bonds to both His291 and Ser365 (left panel, hydrogen bonds denoted with *). Leflunomide (bottom right panel) docked in a similar position but is only able to form a hydrogen bond with His291. Benza(a)anthracene (upper right panel) is apolar and so cannot form hydrogen bonds to these residues. We note that although Thr289 and Gln383 also contribute a polar region to the binding pocket, only 2-(1'H-indole-3'-carbonyl)-thiazole-4-carboxylic acid methyl ester (ITE) and Leflunomide docked with polar groups that could possibly form hydrogen bond interactions to these residues.

**Figure 4:**
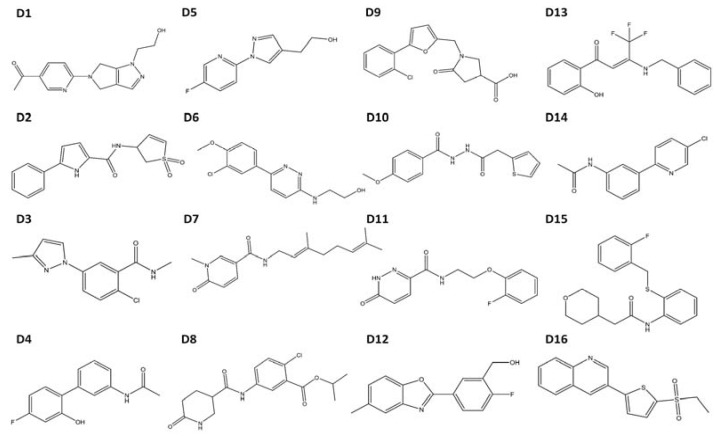
Structures of compounds selected for experimental testing. Compounds **D1**–**D16** scored highly during VLS and also displayed a TCDD-like binding pattern.

Not surprisingly, the compound from the benchmarking analysis with the best docking score was TCDD (−24), for which the model was optimized to interact with, establishing a hydrogen bond with the side chain of Ser365 (Figure 2c, Table 1). The side chain of His291 on the opposite side of the pocket was also available to hydrogen bond to TCDD depending on the tautomerization state (Figure 2c). From our benchmarking analysis we discovered that about half of the other known agonists we docked—including 6-formylindolo[3,2-b]carbazole (FICZ), indirubin, Cl1023, 2-(1ꞌH-indole-3'-carbonyl)-thiazole-4-carboxylic acid methyl ester (ITE), and leflunomide—exhibited TCDD-like binding interactions, forming a hydrogen bond with either His291, Ser365, or both (Figure 3). This commonality suggested to us that interactions with both His291 and Ser365 could be key to agonist affinity, and indeed mutagenesis studies on the murine AHR have demonstrated that mutation of the homologous residues Ser359 and His285 decreases TCDD binding by ca. 50% and ca. 100%, respectively [14]. Therefore our initial search for AHR agonists was based solely on docking results from VLS with a chemically diverse database of ca. 50,000 drug-like compounds, and we then attempted to enrich our selections by choosing high-scoring molecules which established a hydrogen bond to either His291 or Ser365 (Table 2). We subsequently ordered these compounds (**D1**–**D16**) and tested them experimentally to determine if they were in fact AHR agonists (Figure 4).

**Figure 5:**
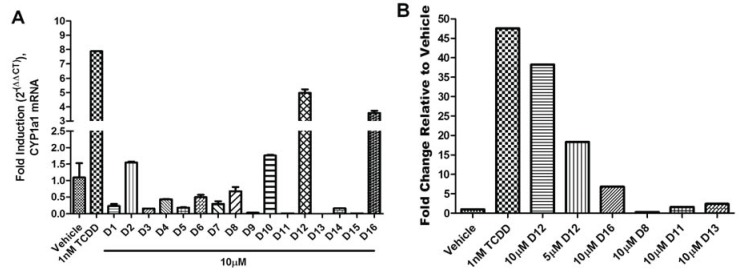
AHR target gene induction of Compounds **D1**–**D16**. (**A**) Quantitative RT-PCR for AHR target gene *CYP1A1* (Cytochrome p450 1A1) in HepG2 cells following exposure to vehicle (DMSO), TCDD (1 nM), and **D1**–**D16** (10 µM) for 12 h. Glyceraldehyde 3-phosphate dehydrogenase (GAPDH) expression was used as a control for normalization of *CYP1A1* expression. Experiments were run in triplicate. (**B**) Human HepG2 cells transfected with the AHR response element (AHRE)/xenobiotic response element (XRE)-luciferase reporter were treated with vehicle (DMSO), TCDD (1 nM), **D12** (5 and 10 µM), and **D16** (10 µM) for 24 h and assayed for reporter gene activity. For comparison representative compounds **D8**, **D11**, and **D13** are shown, which were found to be inactive.

**Figure 6:**
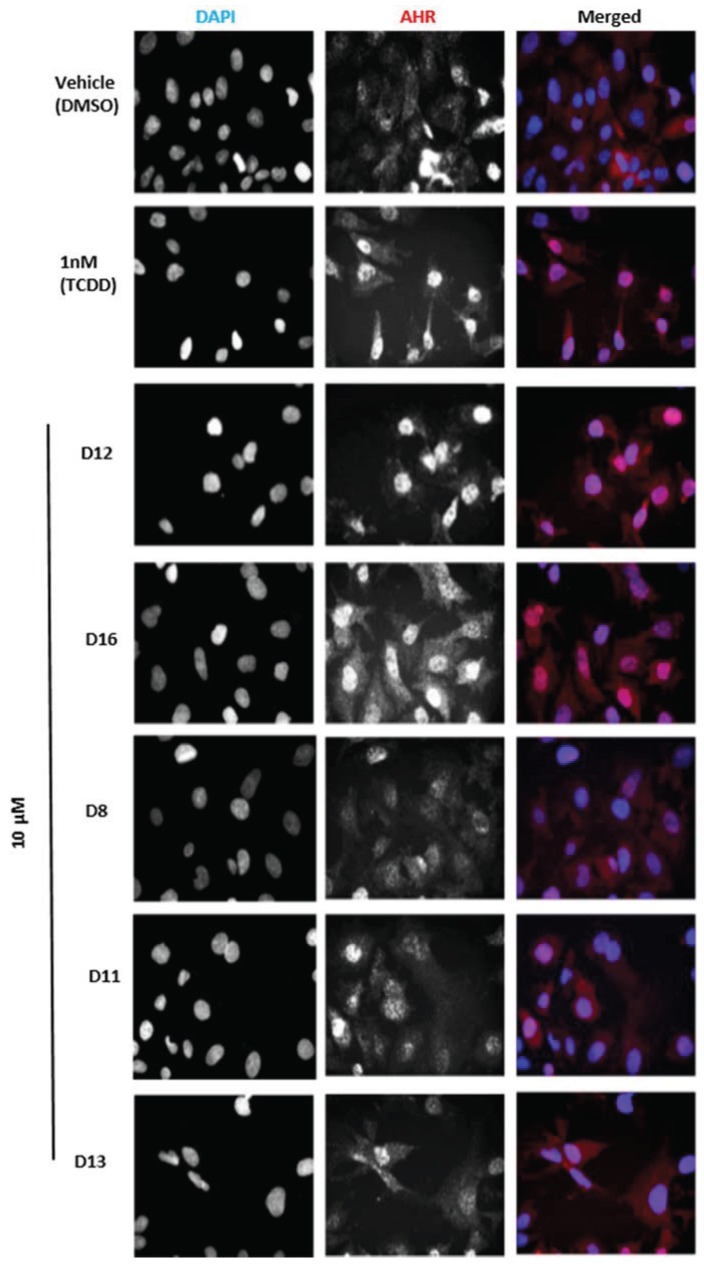
Subcellular localization of AHR in HepG2 cells. Cells were treated with the indicated compounds at 10 µM for 120 min, fixed, and then immunostained with AHR followed by cyanine3 (Cy3)-conjugated secondary antibody. Cells were stained by 4,6-diamino-2-phenylindole (DAPI) to visualize nuclei and were imaged by ImageXpressMICRO wide-field high content imaging system. Experiments were run in triplicate.

**Figure 7:**
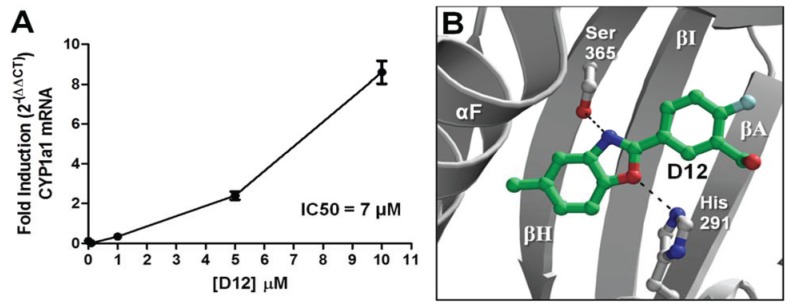
Agonist activity of Compound **D12**. (**A**) For dose-dependency of **D12**, quantitative reverse transcription-polymerase chain reaction (RT-PCR) for AHR target gene *CYP1A1* (Cytochrome p450 1A1) is shown at various [**D12**] following exposure for 12 h. GAPDH expression was used a control. Experiments were run in triplicate. (**B**) The docking orientation of **D12** into the agonist-optimized homology model is shown with the ligand displayed as sticks and colored by atom type as in Figure 2 but with the ligand carbons colored dark green and halogen colored cyan.

The agonist response has two components: the translocation of AHR from the cytosol to the nucleus and induction of AHR target genes. To validate the hits identified from VLS, we first measured each compound’s ability to induce two different AHR target genes in human hepatoma cells (Figure 5). Out of the sixteen compounds screened, **D12** and **D16** were found to activate the endogenous AHR target gene *CYP1A1* (Figure 5a) and AHR-dependent reporter gene (Figure 5b). To determine if this induction was indeed due to the translocation of AHR to the nucleus, we treated human hepatoma cells with Compounds **D1**–**D16** and analyzed AHR localization through immunostaining. We found that the two hits **D12** and **D16** significantly promoted nuclear translocation of endogenous AHR from the cytosol (Figure 6). Thus, both Compounds **D12** and **D16** elicited the characteristic agonist response, and were confirmed to be AHR agonists. The stronger of the two agonists was [2-fluoro-5-(5-methyl-1,3-benzoxazol-2-yl)phenyl]methanol (**D12**), and was found to activate *CYP1A1* in a dose- dependent manner with an IC_50_ of 7 μM (Figure 7a). In our VLS, this compound docked into the AHR PAS-B binding pocket with a score of −26.02 and has two hydrogen bond accepting groups interacting with both His291 and Ser365, similarly to TCDD (Figure 7b, Table 1).

### 3.2. Molecular Dynamics Simulations

Using our agonist-optimized model as a template for the starting coordinates, we conducted in-solution molecular dynamics simulations with AMBER12 [31] to study the differences between the agonist-bound and antagonist-bound forms of AHR PAS-B. We simulated three different versions of our agonist-optimized model: *apo* (no ligand in the binding pocket), agonist (TCDD)-bound, and antagonist (GNF351)-bound, and the simulations came to equilibration by 50–60 ns. In our analysis we focus on the conformations observed at the end stages of simulation, and to distinguish these equilibrated molecular dynamics models from the homology models discussed in the text, we will refer to them as *apo*^MD^, agonist^MD^, and antagonist^MD^.

**Figure 8:**
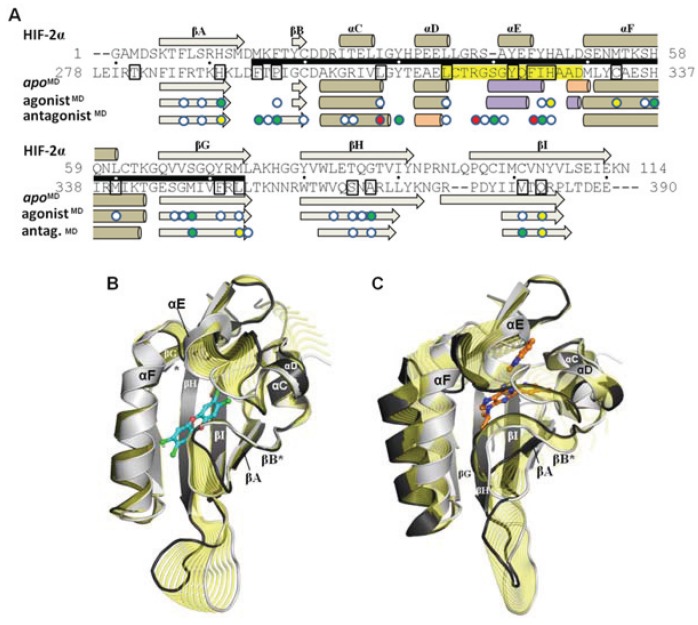
Influence of agonist and antagonist binding on AHR PAS-B structure. (**A**) A structure-based sequence alignment is shown between HIF-2α and the equilibrated AHR molecular dynamics models. The standard secondary structure elements of PAS-B domains are indicated for HIF-2α and the AHR models, with grey arrows depicting β-strands, tan cylinders as α-helices, pink as 3_10_-helices, and purple as pi-helices. Every ten residues are denoted with (.) for the sequence of AHR and residues which have been shown through mutagenesis to be important for agonist binding are noted in black boxes [13,18]. The region of the murine AHR PAS-B that was shown to interact with HSP90 is denoted with a black bar. Colored circles indicate residues contacted by the bound ligands at 50 ns of simulation time, with white indicating ≥1 Å^2^ buried, green for ≥10 Å^2^ buried, yellow for ≥20 Å^2^ buried, and red for ≥30 Å^2^ buried. (**B**) A morph between the *apo*^MD^ (light grey) and agonist^MD^ (dark grey, TCDD shown with blue carbons) conformations is shown, with yellow highlighting the structural differences between the *apo* and the bound state. The secondary structural elements are depicted. The βB and βG regions are noted with an asterisk (*) as they exhibit near β-strand geometry. (**C**) A morph between the *apo*^MD^ (light grey) and antagonist^MD^ (dark grey, GNF351 shown with orange carbons).

Although the models evolved over the course of the simulation, the secondary structure elements were generally retained and the equilibrated models were highly similar to the HIF-2α NMR template from which the starting coordinates of the homology model were derived (Figure 8a). Over ca. 60 Cα atoms, the *apo*^MD^ model differed by ca. 1 Å rmsd from HIF-2α and the agonist^MD^ and antagonist^MD^ models differed by ca. 1 Å rmsd from *apo*^MD^. When comparing the three molecular dynamics models at equilibrium it is apparent that conformational changes occurred during the simulation as a result of the bound ligands, and residues which had substantial contact with the ligands experienced the largest conformational perturbations (Figure 8a). The *apo*^MD^ model experienced shifts mostly at loop regions and at the *N*- and *C*-terminus (Figure 8a). The *apo*^MD^ and agonist^MD^ models are generally similar to each other, with the agonist-bound form having lost some of the helical element of αE (Figure 8b). In contrast, antagonist^MD^ shows substantial rearrangements in the 307–329 region, with αE completely disrupted and αD shortened and converted to a 3_10_-helix (Figure 8c).

### 3.3. Conformational Dynamics Prior to Equilibration

**Figure 9:**
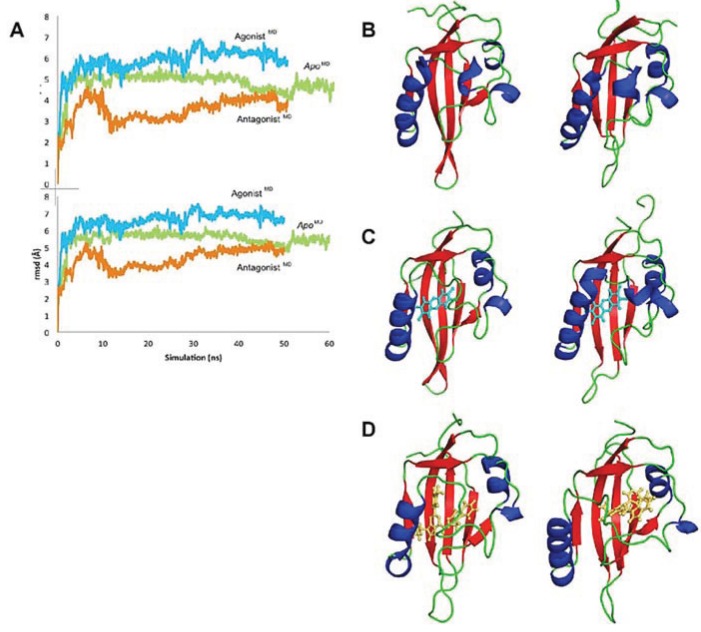
Evolution of the AHR PAS-B structures over time. (**A**) The rmsd at every 100 frames is shown for the backbone atoms of *apo* (green), agonist-bound (cyan), and antagonist-bound (orange) simulations in the top panel; the rmsd for all protein atoms is shown in the lower panel. The equilibrated agonist^MD^ model deviates from the equilibrated *apo*^MD^ by 0.9 Å (69 Cα atoms) and from the HIF-2α PAS-B structure by 1.1 Å (68 Cα atoms), and the equilibrated antagonist^MD^ model deviates from the *apo*^MD^ by 1.1 Å (55 Cα atoms) and 1.2 Å (61 Cα atoms) from HIF-2α. (**b**–**d**) Conformation changes during the course of simulation are shown. The structure at 0 ns is shown left and at the end of the simulation is shown right for *apo* (**B**), agonist-bound (**C**), and antagonist-bound (**D**) simulations. The models are colored by secondary structure: helices, blue; strands, red; loops, green and the ligands are shown as cyan sticks (TCDD) or orange sticks (GNF351)

**Figure 10:**
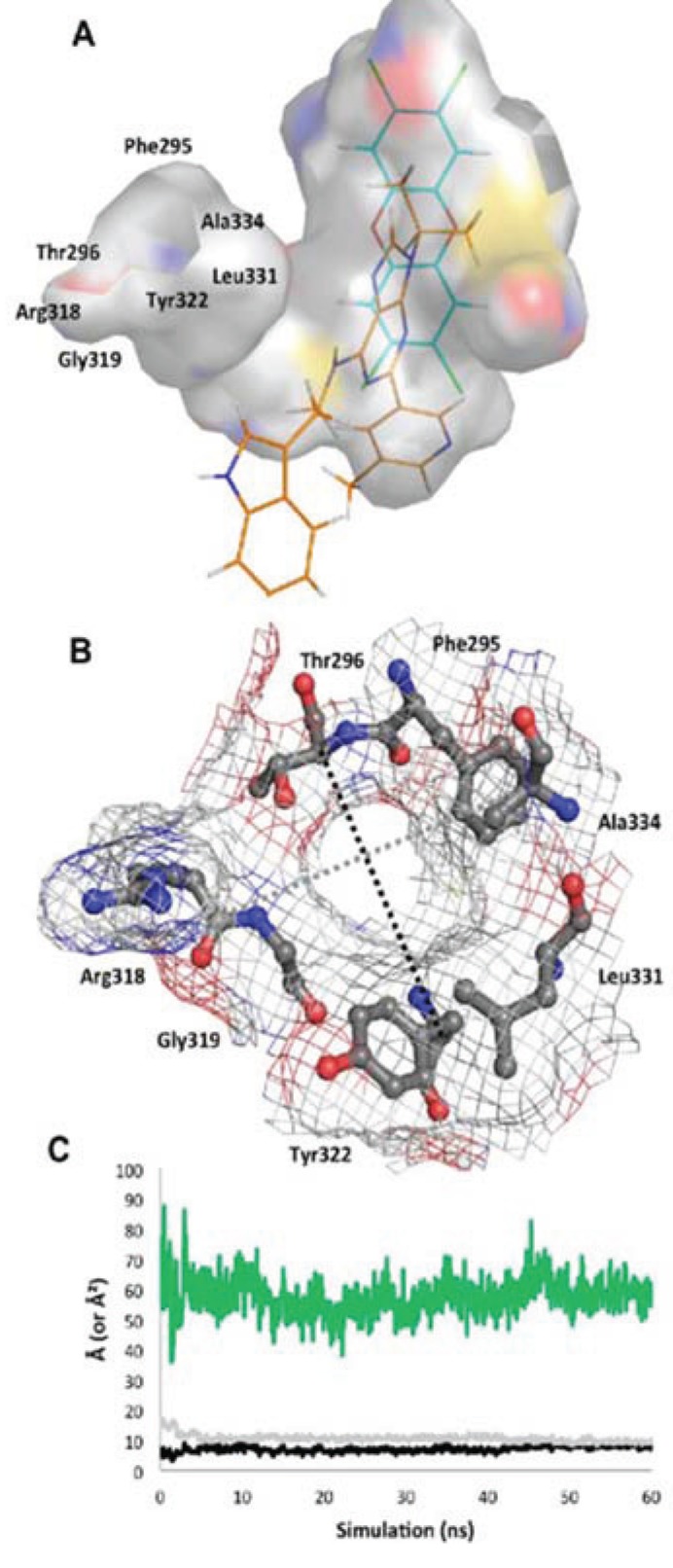
Structure of the *apo* binding pocket. (**A**) The environment of the ligand cavity is shown for *apo*^MD^ (at 60 ns) colored by atom: carbon, grey; oxygen, red; nitrogen, blue; sulfur, yellow with the pocket area calculated as in Figure 2. The ligands of the agonist^MD^ (cyan) and antagonist^MD^ (orange) models are superimposed as lines. (**B**) Seven residues constituting the entrance to the binding site are noted. The pocket entrance is shown, with delineating residues displayed as sticks and surface displayed as mesh colored by atom. (**C**) The approximate size of the pocket entrance over time is shown, estimated by plotting in Å the molecular surface gap (distance between atoms subtracted by molecular surface) between the Cα atoms of residues Ala334 and Arg318 (grey line in b) and Thr296 and Tyr322 (black line in b); the area of the ellipse that would be formed by these two distances is plotted in green in Å^2^.

We analyzed the conformational changes during the progression toward equilibrium to gain insight into the mechanics of the domain (Figure 9a) [32]. The *apo*^MD^ changed little over time, including a shift in the positions of the αE and αF helices prior to 20 ns (Figure 9). The shortening of the βH and βI to coil occurred later in the simulation, as did the slight repositioning of the αC helix. Given that the ligand-binding cavity is proposed to be in the core of the protein [6,13], we were interested to know how the *apo* cavity would appear after solvation and in the absence of its chaperone partner HSP90. As the *apo*^MD^ simulation progressed, the only entrance to the binding pocket, delineated by residues from the βA-βB loop, αE, and αF, shifted from slightly elliptical to circular, becoming stable in a “closed” conformation by ca. 5 ns with a narrow entrance opening of only ca. 50 Å^2^ (Figure 10). Though the binding site in this state would not be accessible to a ligand without additional conformational changes, the pocket interior did not collapse and was roughly the same size and shape as in the agonist^MD^ (Figure 10).

**Figure 11:**
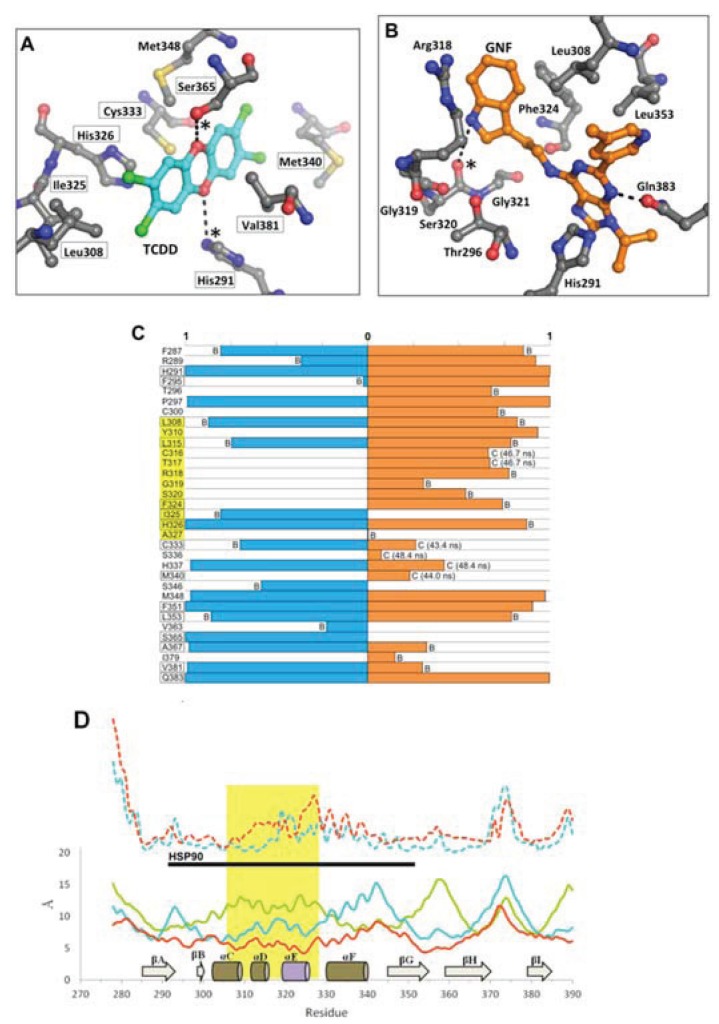
Environment and interactions of the bound ligands. (**A**) The environment of the ligand-binding cavity is shown for the equilibrated agonist^MD^ model (at 50 ns) colored by atom: carbon, grey; oxygen, red; nitrogen, blue; sulfur, yellow. TCDD is depicted with sticks (with carbon atoms colored in cyan); active site residues that form transient hydrogen bonds with the buried polar groups of the ligand are noted with (*). The important interacting residues of the bound TCDD are shown, and residues which have been investigated via mutagenesis and found to influence TCDD binding are noted in black boxes [13,18]. (**B**) As in panel A, the active site cavity is shown for the equilibrated antagonist^MD^ model (at 50 ns). (**C**) Residues contacting the ligand (within 4 Å) during the final 10 ns of simulation are shown with the percentage of contact time noted for agonist^MD^ (blue) and antagonist^MD^ (orange). For those with less than 90% contact time over the final 10 ns, we note whether this is due to fluctuation from being on the pocket boundary (denoted with “B”) or due to a conformational shift (denoted with “C”), for which we also note the time of occurrence. (**D**) Shown is a plot of root mean square fluctuation of Cα atoms to illustrate structural dynamics for *apo*^MD^ (solid green) agonist^MD^ (solid cyan), antagonist^MD^ (solid orange) as calculated by VMD [33] during the last 5 ns of simulation. The Cα shifts between *apo*^MD^ and agonist^MD^ (dashed cyan) and between *apo*^MD^ and antagonist^MD^ (dashed orange) are also shown on the same scale, but for clarity their values are adjusted +20 Å. The 307–329 segment is highlighted in gold, with the secondary structure of the *apo*^MD^ given for reference, and the regions interacting with HSP90 in the murine system are noted by the black bar.

In the agonist^MD^ model, the αE helix was initially disrupted but was mostly regained by the end of the simulation, although in a slightly shifted position (Figure 9). The *N*-terminal end of the αF helix was also disrupted at the beginning of the simulation, but was recovered and actually converted to a pi-helix, which contributed to a constriction of the binding pocket around the ligand. As seen in the close similarity between 0 ns and 50 ns time points, the TCDD shifted very little over the course of the agonist^MD^ simulation, an indication that the conformation of the starting model was energetically favorable (Figure 9). At equilibration, the entrance canal is closed off and the shape adopted by the pocket fits the ligand tightly, with ca. 230 Å^2^ contact area between the ligand and protein (Figure 11a). In our VLS we hypothesized that hydrogen bonds from Ser365 and/or His291 may be a fingerprint for agonist binding, as these interactions were common to many of the docked agonists (Figure 3). In the agonist^MD^ simulation we observed that Ser365 and His291 side chains formed transient hydrogen bonds with the ligand and were at least in contact with the ligand throughout the entirety of the simulation (Figure 11a). A binding profile over the final 10 ns of simulation revealed that the 20 residues contacting the ligand correlate well with positions that have been demonstrated to impact TCDD binding in mutagenesis studies (Figure 11b)[13,18].

Of the three simulations, the antagonist^MD^ model exhibited the most substantial conformation changes over time. The αE helix was disrupted during pre-production molecular dynamics and was never recovered, and the αF helix was seen to become distended from the protein core (Figure 9d). The binding profile is also quite different for antagonist^MD^ (Figure 11). Unlike TCDD, however, to our knowledge no experimental analysis has yet been done to determine what residues are important for binding GNF351. The GNF351 binding profile may therefore serve as an initial analysis to provide guidance for future mutagenesis studies directed at probing antagonist interactions (Figure 11). Overall, the GNF351 had much more extensive contact with residues of the 307–329 region than did TCDD, and consequently the antagonist^MD^ exhibited an altered chain path for the entirety of Leu308-His326 (Figure 11). For both the ligand-bound simulations we also observed considerable alterations in the dynamics of the domain, with the 307–329 segment and the βG-βH regions more stabilized relative to *apo*^MD^ (Figure 11d). In summary, these simulations showed the agonist and antagonist-bound forms of AHR to undergo changes in structure and dynamics. The antagonist required the 307–329 segment to adapt by flexing to a greater degree than when an agonist was bound, and these rearrangements caused substantial distortions to the domain’s structure.

## 4. Discussion

### 4.1. Utility of the Agonist-Optimized Model for Virtual Ligand Screening

VLS is a useful tool that can guide the ranking and selection of potential ligands by efficiently screening thousands of compounds *in silico* and thus, complement current HTS techniques [31]. However, the degree of success of this approach is heavily dependent upon the quality of the model, especially in the absence of an experimentally resolved structure [22]. In this regard, our previous *apo* model was able to screen a database of natural compounds for agonists, but the constricted binding pocket was only able to identify agonists that possessed a flavanoid-like chemical scaffold [6]. We therefore sought to generate a homology model that more closely resembled the agonist-bound state with the anticipation that this would improve the ability to identify increasingly diverse compounds. We constructed models using two different methods: (a) using a *holo* template structure, and (b) docking a strong, well-characterized agonist into the binding pocket of our previous *apo* model and optimizing the pocket to accommodate the ligand. When we tested our models’ ability to distinguish known agonists from random compounds we found that the agonist-optimized model performed better, identifying fourteen out of sixteen true agonists. We therefore continued our analysis using only the agonist-optimized model. In our previous study using the *apo* model we were able to dock TCDD but the binding pocket allowed for only binding energy calculations to be obtained [6]. Using the agonist-optimized model, however, we were able to dock and obtain a docking score for TCDD and each compound, which represents a more accurate prediction for binding because the ICM docking score function accounts for additional parameters including conformational sampling and ligand flexibility [22].

Following the successful screening of known agonists, we probed the agonist-optimized model to further assess its utility. We noted that several inactive compounds were ranked above the threshold value (Table 1). One possibility is that these compounds are in fact binders but do not induce the agonist response, and are antagonists or selective modulators. Thus, we performed a subsequent docking analysis of a small database of well-characterized AHR antagonists and AHR selective modulators (Table 3). We found, however, that such compounds docked with poor scores that were well below the threshold value, showing that the model had some capability of discriminating agonists from antagonists (Table 3). This leads us to hypothesize that the inactives scored highly because they are the flavonoids that were first identified through VLS in our 2009 study using the *apo* model [6], and therefore could be prone to score well in our agonist-optimized model. Still, despite that some inactives scored above the threshold, the six active flavonoids [6] ranked in the top 1% list (Table 1).

**Table 3:** Docking analysis of antagonists and selective modulators.

Compound [ref]	Score
CH223191 [12]	−5.01
DMNF [34]	−2.97
SGA360 [5]	−2.07
GNF351 [5]	+

+: docking score > 0.

When we used the agonist-optimized model to screen a database of 50,000 diverse chemical compounds, sixteen high-scoring compounds emerged as potential agonists based on a combination of docking ranking and structural interactions common to known agonists (Table 2 and Figure 3). Of these, **D12** and **D16** were confirmed to be novel agonists in three separate assays (Figure 5, Figure 6, and Figure 7), with the strongest agonist **D12** exhibiting a moderate agonist IC_50_ of 7 μM (Figure 7a). In addition, we found that neither **D12** nor **D16** showed any evidence of antagonism when co-treatments with TCDD were conducted (SI Figure 1). Although both **D12** and **D16** docked in a similar orientation as TCDD, hydrogen bonding to Ser365 and His291, they possess quite distinct structures and deviate even more so from a flavanoid chemical scaffold than TCDD. This demonstrates that the agonist-optimized model can indeed be applied to search for agonists with non-flavanoid chemical scaffolds, and represents an improvement in utility. A next-step in searching for agonists with more diverse chemical scaffolds would be to select a series of compounds for experimental testing which were ranked above the threshold value but did not dock in a TCDD-like manner. Overall, based on these results we propose that ligand-guided optimization may be a generally useful technique for the generation of AHR homology models for the purpose of VLS. Ligand-optimization could provide a useful alternative for the generation of models based on ligand-bound templates. An example would be the generation of an AHR PAS-B antagonist-bound model, for which no antagonist-bound experimental structures exist that could serve as templates. In fact, similar strategies as reported here that combine homology modeling and molecular simulations have proven to be a successful method for targeting specific GPCR ligand-binding domain conformations [35,36]. Such computational techniques may help to improve database enrichment for NIH ToxCast/Tox21 HTS [37,38].

### 4.2. Evidence of Ligand-Induced Structural and Dynamic Changes to AHR PAS-B

Without an experimentally determined structure we are currently limited to modeling and computational techniques for investigating the structural properties of the AHR PAS-B domain. An important and as-yet undetermined aspect of AHR function is how agonists and antagonists induce distinct cellular responses. In the absence of experimentally resolved structures, it is possible for homology models which have been validated with successful molecular docking of known ligands, such as done here, to be useful for molecular simulations [39]. To our knowledge, molecular dynamics simulations of the ligand-bound AHR PAS-B have never been conducted, and we therefore conducted simulations in the 50 ns range to see what induced-fit conformational changes occur.

The fact that the AHR PAS-B can bind ligands of diverse sizes, shapes, and polarity (e.g., TCDD, leflunomide, benza(a)anthracene, GNF351), suggests the existence of adaptable structural components in the ligand-binding domain. In our simulations, we observed that the region spanning approximately residues 307–329 (αD-αF) adopted different conformations depending on whether the binding pocket was *apo*, agonist-bound, or antagonist-bound (Figure 9). This segment adapted to accommodate the different ligands, with αE unraveling to provide extra volume to the pocket. In both ligand-bound simulations the structure of the 307–329 segment rearranged and substantial and distinct conformation changes occurred when the antagonist was bound. We also found that this region was quite dynamic in the *apo* form but became stabilized when either the agonist or antagonist were bound. Other experimentally determined PAS-B domains structures show the same αD-αE region to exhibit conformational variability [40,41]. Specifically, a deuterium exchange analysis of HIF-2α PAS-B [20] has found that the region possesses high local dynamics when in the *apo* form (implied by poor protection factors) and is stabilized when a ligand is bound (protection increasing for parts of the segment), as we observed in our simulations (Figure 11d). Although the simulations reported here are just 50 ns long and provide a first-look at human AHR structural mechanics, the results appear to be in line with experimental evidence derived from homologues. This suggests that the conformational and dynamic shifts we observed are plausible representations of the domain’s true properties. One *caveat* to this, however, is that the initial docking of the antagonist GNF351 scored poorly (Table 3) which implies that the starting conditions for the antagonist-bound simulation were not ideal. On the other hand, the poor docking score could also be interpreted as the necessity for structural rearrangements to occur for the domain to bind the antagonist.

### 4.3. The 307–329 Segment as a Structural Switch

The results of our simulations lead us to propose two hypotheses regarding AHR function. First, it has been shown that AHR will not bind the agonist TCDD without the association of HSP90 [1]. This suggests a very different behavior from HIF-2α, in which a dynamic transition between open and closed conformers allows for the entry of ligands into the buried cavity [20]. We observed the entrance to the binding pocket of the AHR *apo* form to be shrunken and inaccessible for the duration of the simulation (Figure 10), therefore in agreement with the necessity of the HSP90 interaction for ligand binding. The AHR-HSP90 interacting region has been mapped to the murine AHR and includes the 307–329 segment (Figure 8a and Figure 11d) [1], so it can be inferred that the conformation of this section is an important component of the AHR-HSP90 interface. This leads us to suggest that a primary role of the AHR-HSP90 interaction in the *apo* state is to hold the 307–329 segment of AHR “open” so that the entrance to the pocket is accessible for ligands. Second, alterations to the structure and dynamics of the 307–329 segment would be expected to directly impact the interface with HSP90. We suspect that these conformational shifts promote translocation for agonists, but that the severe distortions induced by an antagonist alter the AHR-HSP90 interface and inhibit translocation, thereby resulting in the observed antagonism of GNF351. In fact, during the preparation of this manuscript recent results published examining the mouse AHR [42] seem to support that the structural packing of the 307–329 segment is indeed critical for the AHR-HSP90 interface, as mutation of two residues that are conserved in both human and mouse AHR-Phe324 and Ile325—impacted ligand binding and decreased AHR-HSP90 complex formation [42].

In summary, these 50 ns molecular dynamics simulation lead us to view the 307–329 region as a structural switch that operates through perturbing a sensitive AHR-HSP90 complex, with three distinct conformations corresponding to three biological responses: (i) in *apo* form, the conformation is compatible with the association with HSP90 and other chaperones, and the complex remains latent in the cytosol; (ii) upon agonist binding, the conformation shift causes structural rearrangements that promote AHR translocation to the nucleus; and (iii) upon antagonist binding, the conformation promotes premature dissociation of HSP90 or causes structural changes that inhibit AHR nuclear translocation. Further experimental studies will be required to test the accuracy of these predictions.

## 5. Conclusions

This study has provided insights into how the human AHR PAS-B domain interacts with structurally diverse ligands. We have proposed that ligand-specific optimizations of AHR PAS-B homology models may be a generally useful method for improving utility for VLS when searching for certain classes of AHR ligands. Our agonist-optimized homology model of human AHR PAS-B was used to describe a His291/Ser365 binding pattern of known AHR activators and identify new AHR agonists by VLS. This model was then utilized to investigate conformational changes in the presence of an AHR agonist (TCDD) and antagonist (GNF351) by means of molecular dynamics. In our simulations we found that the 307–329 segment exhibited substantial shifts in conformation and dynamics depending on whether the binding pocket was unoccupied, agonist-bound, or antagonist-bound. These distinctive changes suggest this segment is a switch that, through altering the AHR-HSP90 interface, initiates either agonist or antagonist biological responses. Further, it may be possible for the antagonist-bound AHR conformation to be used to screen for new AHR antagonists. The studies described here will aid in the future discovery of novel AHR modulators and complement current HTS techniques.

## Supplementary Files

Supplementary File 1

## Abbreviations

AHR: Aryl hydrocarbon receptor
TCDD: 2,3,7,8-tetrachlorodibenzo-p-dioxin
GNF351: *N*-2- (1H-indol-3yl)ethyl)-9-isopropyl-2-(5-methylpyridin-3-yl)-9H-purin-6-amine
**D12**: [2-fluoro-5-(5-methyl-1,3-benzoxazol-2-yl)phenyl]methanol
VLS: virtual ligand screen
PAS: Per/ARNT/Sim
ARNT: AHR nuclear translocator
HSP90: heat shock protein 90
HIF-2α: hypoxia inducible factor 2α
DMSO: dimethyl sulfoxide
Ca.: Circa
