# Supplementary material for: A Structural Switch between Agonist and Antagonist Bound Conformations for a Ligand-Optimized Model of the Human Aryl Hydrocarbon Receptor Ligand Binding Domain
Source: Biology (Basel). 2014 Oct 17;3(4):645–69. doi: 10.3390/biology3040645 (PMC4280506; doi:10.3390/biology3040645)
Supplement: Supplementary File 1 [file biology-03-00645-s001.docx]

**Supplemental Material**

**Figure S1.** Co-treatment of TCDD and compounds **D12** and **D16**. Levels of *CYP1A1* mRNA measured by quantitative real-time PCR following a 1 hour pre-treatment with 50 pM TCDD and a subsequent 8 h co-treatment with 10 µM of the indicated compound. The full antagonist CH223191 was included as a control.
